# Knowledge, attitude, practice towards breast self-examination and associated factors among women in Gondar town, Northwest Ethiopia, 2021: a community-based study

**DOI:** 10.1186/s12905-022-01764-4

**Published:** 2022-05-14

**Authors:** Kibret Asmare, Yeneabat Birhanu, Zerko Wako

**Affiliations:** 1 Department of Surgical Nursing, School of Nursing, College of Medicine and Health Sciences, University of Gondar, Gondar, Ethiopia; 2 Department of Medical Nursing, School of Nursing, College of Medicine and Health Sciences, University of Gondar, Gondar, Ethiopia

**Keywords:** Attitude, Knowledge, Practice, Breast self-examination, Factors, Ethiopia

## Abstract

**Introduction:**

Breast cancer is the most common type of cancer among women, particularly in low and middle-income countries. Breast self-examination is one of the non-invasive methods of screening in which a woman looks at her breast for any abnormal findings like lumps, distortions, or swellings. Despite, realized effects of breast self-examination in detecting breast cancer earlier, the vast majority of the cases still present with an advanced stage.

**Objective:**

This study aimed to assess knowledge, attitude, and practices toward breast self-examination and associated factors among women in Gondar Town, Northwest Ethiopia, 2021.

**Methods:**

A community-based cross-sectional study was conducted on women living in Gondar town. A simple random sampling method was used to select 571 participants. Interviewer administered questionnaires were used for data collection. Data was entered into Epi-data version 4.6 and exported to Statistical Package for Social Science (SPSS) version 25 for analysis. Multivariate logistic regression was used where a *p*-value < 0.05 was used to identify variables significantly associated with the outcome variable.

**Result:**

From the total of 571 women, about 541 participants were involved in the study with a response rate of 94.7%. Of these, 56%, 46% and 45.8% of women had adequate knowledge, favourable attitudes, and performed breast self-examination (BSE) respectively. Women College and above AOR: 3.8 (95% CI: 1.43–10.14) and spouses College and above AOR: 3.03 (95% CI: 1.04–8.84), Women College and above AOR: 4.18 (95% CI: 1.59–10.92) and history of breast cancer AOR: 6.06 (95% CI: 2.19–16.74) and knowledge level AOR: 2.67 (95% CI: 1.18–6.04) were significantly associated with knowledge, attitudes, and practices towards breast self-examination respectively.

**Conclusion:**

The findings of this study were considerable for inadequate knowledge, unfavourable attitude and poor practice towards BSE among women. Emphasis should be made on boosting the knowledge, attitude, and practice of the women toward breast self-examination and strengthening the implementation of comprehensive, systematic, and continuous BSE educational programs that were recommended along with a breast cancer awareness campaign.

## Introduction

Breast cancer is a type of malignant tumor that starts in the cells of the breast and commonly occurs in women [1]. This abnormal cell could destroy healthy tissue and then, spread beyond its usual boundaries [2]. Non-communicable diseases (NCD) including breast cancer considered not prevented, which is a noticeable common false perception of the community but it is a preventable cause of morbidity, disability, and mortality [3]. The most common risk factors of breast cancer are being a woman and getting ≥ 50 years old, having dense breasts, having a family history of breast cancer, early menstruation, and late menopause, late pregnancy, being on birth control pills, race, atypical hyperplasia of the breast, and previous treatment using radiation therapy [4, 5]. Lifestyle factors are obesity, lower amounts of physical activity, alcohol, and foods like high fats and low fiber diets [5]. Evidence suggested that; getting regular physical activity, staying at a healthy weight, limiting the amount of alcohol drink, avoiding the use of postmenopausal hormone therapy, breastfeeding, eating more fruits, vegetables, and fewer animal fats is linked with many health benefits and lower the risk of breast cancer [5]. Besides, it not only affect the breast tissue, but also spread to other part of the body organs like liver and lungs [6, 7].

According to global cancer statistics; breast cancer accounts for about 25% and 15% of total incidence and death respectively [8, 9]. In American an average risk of a woman of developing a breast cancer was 13% which is equivalent to one in every eight women had a chance of developing in their life time [10]. It is an important public health issues, not only in the developed world but also in resource limited nations [11]. It is the most frequent cause of death among women and estimated that 70% of all breast cancer cases worldwide were reported in low and middle-resource countries [12]. The incidence of breast cancer accounts for 22.6% of all cases of cancer in Ethiopia [13]. Breast self-examination (BSE) is one of the non-invasive methods of screening in which a woman looks at her breast for any abnormal findings like lumps, distortions, discharges or swellings with an intention to detect it early for early initiation of treatment and better chance of survival for breast cancer patients [14, 15].

Knowledge, attitude and practice of BSE have been reported in different countries. In regards to BSE knowledge 22.7% in Vietnam [16], 63.8% in Turkey [17], 41.5% in Libya [18], 34% in Sudan [19], 78% in Cameroon [20], 25.6% in Addis Ababa [21], 34.2% in Arba Minch [22], 43.1% in Jima [23], and 55.5% in Adwa town [24] had an adequate knowledge about breast self-examination. As per women’s attitude 64.01% in Saudi Arabia [25], 87.2% in Pakistan [26], 74.9% in Libya [18], 53.4% in Addis Ababa [21], and 46.3% in Adwa town [24] had a favorable attitude towards BSE. Considering practice towards BSE 43% in Saudi Arabia [27], 24.9% in Pakistan[26], 21% in Oman [28], 15.8% in Vietnam [16], 8.5% in turkey [17], 37.6% in Ghana [29], 38.5% in Cameroon [30], 39.2% in Egypt [31], 12.1% in Libya [18], 20.6% in Sudan [19], 13.3% in Arba Minch [32], and 18.6% in Addis Ababa [33] had a good practice of breast self-examination.

Factors affecting knowledge of the BSE were reported from the different countries. These are age, marital status, level of education, information of BSE, medical background, access to internet, source of information, and level of income. However, only the education levels and medical background for attitude towards BSE [16, 18, 26, 31, 33, 34]. Practice factors are almost similar to that of knowledge other than health education on breast cancer, knowing BSE techniques, occupation, and family history of breast cancer were related to women’s knowledge, attitude and practice towards BSE [16, 18, 33–38].

BSE is the only feasible approach that is cheap and easily applied method across wide population. Its ultimate purpose is early detection and treatment. Despite, its importance as an early detection strategy, poor knowledge of women has been a major obstacle. So, amplifying women’s knowledge, attitude, and practice towards BSE through creating breast cancer awareness campaign [39–43].

In our study area, there is a lack of evidence on women’s knowledge, attitude, and practice towards breast self-examination (BSE). Therefore, this study was aimed at assessing women’s knowledge, attitude, and practice on breast self-examination and thereby generates appropriate information that can be used by the central Gondar zonal health office and non-governmental organizations in the prevention and interventions of breast cancer.

## Methods and materials

### Study design, and period

A community-based cross-sectional study was conducted from April to May 2021.

### Study setting

The study was conducted at Gondar Town, Northwest Ethiopia. Gondar Town is located 748 km from Addis Ababa the capital city of Ethiopia and 180 km from Bahir Dar capital city of Amhara regional state. The town has 6 sub-cities with 25 Kebele and 127,115 houses. Gondar Town is one of the ancient and densely populated towns in Ethiopia. According to the central Gondar zonal health office 2021 GC report, Gondar has a total population of 432,191 of whom 215,663 are men and 216,538 women. Nowadays the town is growing and it had 8 health centers, more than 15 private medical clinics, one private primary hospital, and one Comprehensive Specialized Hospital serving about 5 million populations.

### Source population and study population

All adult women who are living in Gondar town were the source population. All women who are living in Gondar town during the data collection period were considered as the study population.

### Inclusion and exclusion criteria

All women who were 20–70 age group living in Gondar town during the study period were included. Women who are severely ill during data collection time and those who refuse to participate in the study were excluded.

### Sample size determination and calculation

The actual sample size for the study was determined using single population proportion formula with the assumption of 34.2% knowledgeable of BSE [32], 95% confidence interval; (α = 0.05), 5% margin of error, 10% none response rate, and 1.5 design effect.

$${\text{n}} = \frac{{\left( {{\text{Z}}\upalpha /2} \right)2\;{\text{p}}\left( {1 - {\text{p}}} \right) }}{{{\text{d}}2}} = \frac{{\left( {1.96} \right)2 \left( {0.342} \right) \left( {0.658} \right) }}{{\left( {0.05} \right)2}} = 346,$$

where n = is the minimum sample required; P = population proportion, 0.342; d = the margin of error, 0.05; Z = the upper percentile of the normal distribution at 95% CI.

By Multiply 1.5 design effect n = 519, adding 10% non-response rate = 52 and the final sample size would be 571 (Table 1). Sample size calculation on second the objective in the same study showed that only practice was significantly associated with BSE (Table 2).

**Table 1:** Sample size calculation for the dependent and independent variables among women in Gondar town, Northwest Ethiopia, 2021

Variable	Assumption	*P*-value (%)	Sample size	Non-response (10%)	Design effect (1.5)	Reference
Knowledge	CI = 95% margin of error (α = 5%)	34.2	346	381	571	[32]
Attitude		14.5	191	210	315	
Practices		21.3	258	284	426

**Table 2:** Sample size calculation for associated factors with breast self-examination among women in Gondar town, Northwest Ethiopia, 2021

Factors	Assumption	Proportion	Initial sample size	1.5 Design effect	10% Non-response	Reference
Age of the women	CI = 95%	P1 = 23.22%	242	363	400	[32]
	Power = 80	P2 = 39.19%				
Women’s level of education	Ratio 1:1	P1 = 15.81%	136	204	225	
		P2 = 38.46%				
Women’s level of knowledge	Practice of BSE	P1 = 13.28%	138	207	228	
		P2 = 35.02%				

The final sample size was from a single population proportion and the second objective was used the maximal one take from single population proportion knowledge of BSE = 571.

### Sampling technique and procedures

**Fig. 1:**
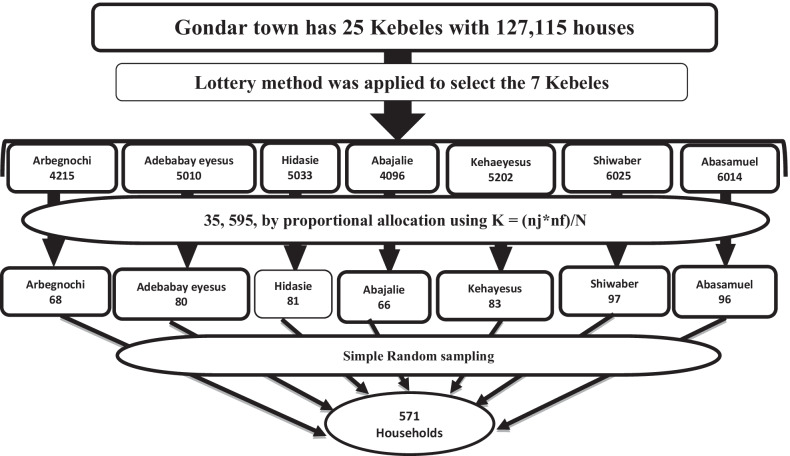
The schematic presentation of sampling procedure to select the study households of women aged 20–70 at Gondar Town, Northwest Ethiopia, 2021 (n = 571)

A multi-stage sampling method was applied for the selection of houses from each Kebele. Firstly, Out of 25 kebeles in the Gondar town, 7 kebeles such as Aribengochi, Adebabayeyesuse, Abajalie, kehaeyesus, Shiwaber, Hidasie, and Aba Samuel kebele were randomly selected by lottery method to represent all kebeles (28% representativeness). Secondly, a systematic sampling technique was used to label the households within each Kebele The proportional allocation formula for each Kebele is $n = \frac{njXnf}{N}$; Were, nf = final sample size, nj = total number of houses each Kebele, N = total number of houses (Fig. 1). Lastly, a simple random sampling was used to select 571 study participants within households using the sampling frame.

### Data collection tools and procedures

The questionnaire was adapted from different works of literature [26, 44, 45]. The questionnaire was first prepared in English, and then it was translated into Amharic language and back to English. It contains four parts such as socio-demographic characteristics (n = 9), knowledge (n = 12), attitude (n = 13), and practice (n = 8) items. Then the data were collected by 5 data collectors using face-to-face interviewer administer questionnaires. The overall supervision was carried out by the principal investigator.

### Operational definition

*Adequate knowledge*: It refers to participants who scored mean and above values 8 from the provided 12 close-ended questions about the knowledge of BSE [46].

*Inadequate knowledge*: It refers to participants who scored below mean values 8 from the provided 12 close-ended questions about the knowledge of BSE [46].

*Favorable attitude*: It refers to participants who scored mean and above values 6 for attitude-related questions towards BSE, which was measured by the provided 12 questions [46].

*Unfavorable attitude*: It refers to participants who scored below mean values 6 for attitude-related questions towards BSE, which was measured by the provided 12 questions [46].

*Good practice*: It refers to those who checked or perform BSE at least once per month just a week after each menses [47].

*Poor practice*: It refers to those who practice BSE other than the correct time in the cycle [47].

### Data quality control

Pretest was done on 5% of the total sample size from the non-selected kebele of the same population before 4 days of actual data collected. Two days of training were given for data collectors and supervisors by investigators on the objectives of the study, ethical considerations, the content of the questionnaire, and how to conduct the interview. Based on the pre-test, questionnaires were edited, and necessary corrections made were on order of questions and that poses participant to confusion. The reliability of the tool on Cronbach’s alpha results knowledge = 0.81, attitude = 0.86, and practice = 0.71. The collected data was checked out for completeness, accuracy, and clarity by the principal Investigators before data entry.

### Data processing and analysis

Data were entered using Epi data version 4.6 software and analyzed using SPSS version 25. Data cleaning and cross-checking were done before analysis. Descriptive statistics were summarized using the mean, and standard deviation. Frequencies and percentages were used in the presented table, figures and text. Multivariate logistic regression analysis were used to identify factors where *p*-value < 0.05 was declared as significantly associated with knowledge, attitudes, and practices of breast self-examination.

## Result

### Socio-demographic characteristics

Out of 571 participants, 541 agree to participate in the study, yielding a response rate of 95%. Nearly half (47.5%) of the participants were within the age range between 20 and 29 years. The minimum and maximum age of the participants was 20 and 69 respectively, the majority of women (87.4%) and 36.8% were orthodox and completed secondary education level respectively. Among the participants, 62.1% and 49.9% were married and housewives respectively (Table 3).

**Table 3:** Socio-demographic characteristics of the women aged 20–70 at Gondar Town, Northwest Ethiopia, 2021

Variables	Response	Frequency	Percent
Age of the women	20–29 years	257	47.5
	30–39 years	153	28.3
	40–49 years	54	10.0
	50–70 years	77	14.2
Marital status	Single	125	23.1
	Married	336	62.1
	Widowed	36	6.7
	Divorced	44	8.1
Religion	Orthodox	473	87.4
	Muslim	54	10.0
	Protestant	13	2.4
	Catholic	1	0.2
Level of education	Illiterate	76	14.0
	Primary school	105	19.4
	Secondary school	199	36.8
	College and University	161	29.8
Occupation	Housewife	270	49.9
	Merchant	60	11.1
	Student	76	14.0
	Governmental employee	81	15.0
	House worker	54	10.0
Husband’s educational level	Illiterate	44	13.1
	Primary	70	20.8
	Secondary	105	31.3
	College and University	117	34.8
Monthly income	< 500 Birr	22	4.1
	500–1000 Birr	36	6.7
	≥ 1001 Birr	483	89.3
History of breast cancer	Yes, I have	35	6.5
	No, I don't have	506	93.5

### Knowledge towards breast self-examination and their information source

**Fig. 2:**
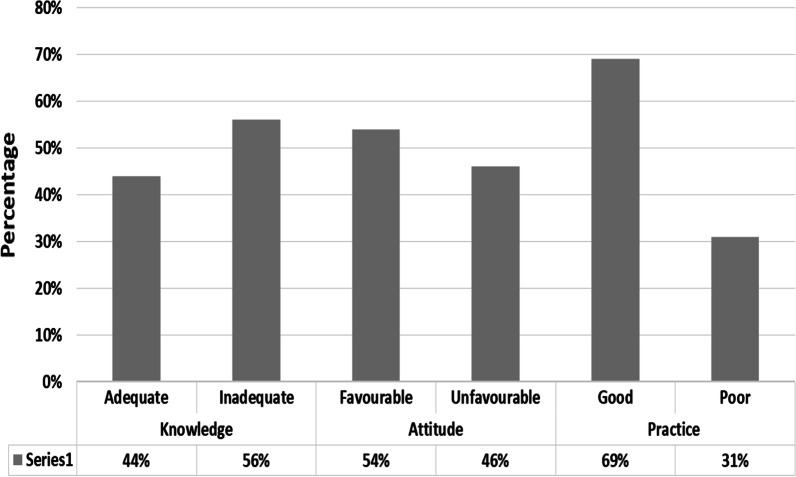
The overall status of knowledge, attitude, and practice towards Breast self- examination among women aged 20–70 at Gondar Town, Northwest Ethiopia, 2021 (n = 571)

Among the respondents, 66% of participants heard about breast self-examination. The majority (56%) (95% CI: 51–61) of participants had adequate knowledge (Fig. 2) and their main source of information was television/radio (43.2%), health institution/profession (30.8%), peer group, and school training (15.6%) and from newspaper and internet (10.2%) (Table 4).

**Table 4:** Knowledge towards breast self-examination among women aged 20–70 at Gondar Town, Northwest Ethiopia, 2021

Questions	Responses	Percentage/frequency
Have you ever heard of breast self-examination before?	Yes	66.0% (n = 357)
	No	34.0% (n = 184)
Where did heard from?	Healthcare provider	28.9% (n = 103)
	Peer group	20.4% (n = 73)
	Television/Radio	38.1% (n = 136)
	Newspaper	12.6 (n = 45)
Does early detection of breast cancer improve chances of survival?	Yes	86.0% (n = 307)
	No	14.0% (n = 50)
Who should perform BSE?	Males only	0
	Females only	81.0% (n = 289)
	Both	19.0% (n = 68)
	Don’t know	0
Is BSE important in the early detection of breast cancer?	Important	68.3% (n = 244)
	Not important	31.7% (n = 113)
	I don’t know	0
How often should breast self-examination be performed?	Weekly	39.2% (n = 104)
	Monthly	54.3% (n = 144)
	Yearly	4.9% (n = 13)
	Don’t know	1.5% (n = 4)
Where did the appropriate place to perform BSE?	While lying in the bed	43.2% (n = 236)
	In front of the mirror	17.6% (n = 96)
	While having a bath	39.3% (n = 215)
Changes in the shape and color of the breast are the signs of breast cancer	Yes	37.0% (n = 132)
	No	63.0% (n = 225)
Lumps in the breast and around the armpit are the signs of breast cancer	Yes	66.4% (n = 237)
	No	33.6% (n = 120)
Nipple discharge and retraction are the signs of breast cancer	Yes	66.9% (n = 238)
	No	33.1% (n = 118)
How do you prefer your breast examination to be done?	By nurse/doctor	65.0% (n = 232)
	By yourself	35.0% (n = 125)
Where will you go, if there are any symptoms of breast cancer	Health facility	99.2% (n = 354)
	Traditional healer	0.8% (n = 3)

### Attitude towards breast self-examination

Among 541 study participants, 46.0% (95% CI: 42–52) had positive attitudes towards breast self-examination, 60.3% of the respondents score breast self-examination as necessary (Table 5).

**Table 5:** Attitude towards breast self-examination among women aged 20–70 at Gondar Town, Northwest Ethiopia, 2021

Questions	Responses	Percentage/frequency
Breast self-examination is necessary	Yes	60.3% (n = 326)
	No	39.7% (n = 215)
Do you think breast self-examination can give you a benefit	Yes	60.1% (n = 325)
	No	39.9% (n = 216)
During BSE makes me feel so funny	Yes	6.3% (n = 34)
	No	93.7% (n = 507)
BSE is not embarrassing to me	Yes	79.7% (n = 431)
	No	20.3% (n = 110)
Doing BSE is not wasting time	Yes	44.9% (n = 243)
	No	55.1% (n = 298)
After doing BSE makes me feel satisfying	Yes	44.2% (n = 239)
	No	55.8% (n = 302)
If there is a lump, I prefer to get treatment from a health institution	Yes	46.2% (n = 250)
	No	53.8% (n = 291)
If I can do BSE once a month, I feel comfortable	Yes	41.8% (n = 226)
	No	58.2% (n = 315)
All women should do BSE	Yes	44.7% (n = 242)
	No	55.3% (n = 299)
I care about my breasts	Yes	44.4% (n = 240)
	No	55.6% (n = 301)
I'm afraid to think about the breast cancer	Yes	87.1% (n = 471)
	No	12.9% (n = 71)
Because I always worry about having breast cancer, I want to do BSE	Yes	39.9% (n = 216)
	No	60.1% (n = 325)

### Practices towards breast self-examination

Among 541 study participants, 248 (45.8%) performing breast self-examination and 31% (95% CI: 25–37%) had good practices of breast self-examination (Fig. 2). From the total respondents, more than half 54.2% (n = 293) of them did not practice BSE. The main reasons for not practicing BSE 175 (57.4%) were not having breast problems and 31.7% (n = 93) don’t know how to breast self-examine (Table 6).

**Table 6:** Practice towards breast self-examination among women aged 20–70 at Gondar Town, Northwest Ethiopia, 2021

Questions	Responses	Percentage/frequency
Have you ever done breast self-examination?	Yes	45.8% (n = 248)
	No	54.2% (n = 293)
If ‘Yes’ when did you perform it?	Just a week after each menses	31% (n = 77)
	When it comes to mind	52.4% (n = 130)
	Anytime during menses	10.9% (n = 27)
	Before menses	5.6% (n = 14)
If ‘No’ then why haven’t you performed it?	Fear of detecting an anomaly	0.3% (n = 1)
	there are no problems with my breast	58.4% (n = 171)
	Not necessary	7.5% (n = 22)
	Too busy	2.0% (n = 6)
	Don’t know how to self-examine	31.7% (n = 93)
How is breast self-examination done?	Palpate with one finger	1.2% (n = 3)
	Palpate with palm and three fingers	98.4% (n = 244)
	I do not know	0.4% (n = 1)
At what age did you start breast self-examination?	20–29 years	56.5% (n = 140)
	30–39 years	28.6% (n = 71)
	40–49 years	12.9% (n = 32)
	50-70 years	2.0% (n = 5)
What time do you normally perform Breast self-examination?	Morning	21.8% (n = 54)
	Afternoon	18.1% (n = 45)
	Evening	60.1% (n = 149)
Where do you usually perform breast self-examination?	In front of the mirror	18.4% (n = 73)
	Lying on the bed	45.8% (n = 182)
	In the bathroom	35.8% (n = 142)
On identifying an abnormality in your breasts, what would you do?	Tell mother	4.4% (n = 11)
	Tell spouse	6.5% (n = 16)
	Consult doctor/nurse	89.1 (n = 221)
	Not do anything about it due to embarrassment	0
	Option for traditional healing	0

### Factors associated with breast self-examination

All independent variables entered into the logistic regression (multivariable logistic regression analysis). Women’s and husbands' education level were significantly associated with knowledge. The age of the women and women’s educational level was significantly associated with the attitude of BSE. History of breast cancer and good knowledge of BSE were significantly associated with performing BSE. Women college and above were about 4 times [AOR: 3.8, (95% CI: 1.43–10.14)] more likely to know of BSE than those who uneducated women. The spouses whose educational level College and above was about 3 times [AOR: 3.03, (95% CI: 1.04–8.84)] more likely to be knowledgeable towards BSE than those who had an illiterate husband (Table 7).

**Table 7:** Bivariate and multivariate analysis of factors associated with knowledge of BSE among women aged 20–70 at Gondar Town, Northwest Ethiopia, 2021

Variables	Knowledge of BSE		*P*-value	COR (95% CI)	AOR (95% CI)
	Adequate	Inadequate			
*Age of the women*					
20–29 years	81	72	1.0	1.0	1.0
30–39 years	66	45	0.293	1.30 (0.80–2.14)	1.12 (0.61–2.06))
40–49 years	28	16	0.210	1.56 (0.78–3.11)	2.12 (0.92–4.86))
50–70 years	25	24	0.815	0.93 (0.49–1.76)	1.21 (0.51–2.89)
*Marital status*					
Single	39	33	1.0	1.0	1.0
Married	128	96	0.658	1.13 (0.66–1.92)	0.51 (0.16–1.68)
Widowed	15	12	0.902	1.06 (0.44–2.57)	1.42 (0.43–4.69)
Divorced	18	16	0.906	0.95 (0.42–2.16)	1.53 (0.53–4.36)
*Level of education*					
Illiterate	9	22	1.0	1.0	1.0
Primary school	21	39	0.834	0.91 (0.38–2.17)	0.67 (0.26–1.71)
Secondary	72	66	0.154	1.74 (0.81–3.75)	1.41 (0.59–3.31)
College& above	98	30	0.000	5.53 (2.49–12.28) **	3.80 (1.43–10.14) **
*Occupation*					
Maid	11	20	1.0	1.0	1.0
Merchant	28	19	0.040	2.68 (1.05–6.85) *	1.56 (0.51–4.71)
Student	26	17	0.036	2.78 (1.09–7.24) *	1.86 (0.65–5.29)
Gov’t employee	53	15	0.000	6.42 (2.53–16.33) **	2.11 (0.65–6.85)
Housewife	82	86	0.175	1.73 (0.78–3.84)	1.38 (0.49–3.90)
*Spouse educational level*					
Illiterate	9	17	1.0	1.0	1.0
Primary	19	17	0.159	2.1 (0.75–5.97)	2.60 (0.83–8.20)
Secondary	32	37	0.304	1.63 (0.64–4.17)	1.94 (0.68–5.54)
College and above	68	25	0.001	5.14 (2.03–13.01) **	3.03 (1.04–8.84) **
*Women monthly income*					
< 500 Birr	7	12	1.0	1.0	1.0
500–1000 Birr	7	16	0.661	0.75 (0.21–2.72)	1.43 (0.36–5.68)
> 1001 Birr	186	129	0.064	2.47 (0.95–6.45)	2.31 (0.81–6.59)
*History of breast cancer*					
No, I don’t have	191	149	1.0	1.0	1.0
Yes, I have	9	8	0.361	1.45 (0.65–3.25)	1.77 (0.71–4.44)

^**^Indicates *P*-value < 0.001 strongly associated, *Indicates *P*-value < 0.05 weakly associated, 1.0 indicates reference

Women College and above was about 4 times [AOR: 4.18 (95% CI: 1.59–10.92)] and secondary school was about 3 times [AOR: 2.80 (95% CI: 1.25–6.29)] had favorable attitudes towards BSE than those women who are illiterate (Table 8).

**Table 8:** Bivariate and multivariate analysis of factors associated with Attitude towards BSE among women aged 20–70 at Gondar Town, Northwest Ethiopia, 2021

Variables	Attitude of BSE		*P*-value	COR (95% CI)	AOR (95% CI)
	Positive	Negative			
*Age of the women*					
20–29 year	109	148	0.015	1.0	1.0
30–39 year	77	76	0.120	1.38 (0.92–2.06)	1.11 (0.61–2.01)
40–49 year	29	25	0.131	1.58 (0.87–2.84)	1.15 (0.48–2.74)
50–70 year	33	44	0.945	1.02 (0.61–1.70)	1.12 (0.47–2.66)
*Marital status*					
Single	52	73	0.062	1.0	1.0
Married	156	180	0.355	1.22 (0.80–1.84)	1.29 (0.39–4.25)
Widowed	20	16	0.140	1.76 (0.83–3.71)	2.52 (0.72–8.78)
Divorced	20	24	0.657	1.17 (0.59–2.34)	1.43 (0.45–4.48)
*Level of education*					
Illiterate	19	57	0.000	1.0	1.0
Primary school	27	78	0.913	1.04 (0.53–2.05)	1.15 (0.48–2.72)
Secondary	94	105	0.001	2.69 (1.49–4.84)*	2.80 (1.25–6.29) *
College and above	108	53	0.000	6.11 (3.31–11.30)*	4.18 (1.59–10.92) **
*Occupation*					
Maid	12	42	1.0	1.0	1.0
Merchant	31	29	0.002	3.74 (1.65–8.47) *	1.79 (0.55–5.92)
Student	39	37	0.001	3.69 (1.69–8.08)**	3.17 (0.11–9.02)
Gov’t employee	51	30	0.000	5.95 (2.72–13.03) **	1.20 (0.35–4.17)
Housewife	115	155	0.006	2.59 (1.31–5.15)*	2.88 (0.96–8.66)
*Spouse educational level*					
Illiterate	15	29	1.0	1.0	1.0
Primary	19	51	0.431	0.72 (0.32–1.63)	0.41 (0.14–1.19)
Secondary	46	59	0.272	1.51 (0.72–3.14)	0.93 (0.35–2.49)
College and above	76	41	0.001	3.58 (1.73–7.44) **	1.19 (0.42–3.38)
*Women monthly income*					
< 500 Birr	10	12	1.0	1.0	1.0
500–1000 Birr	10	26	0.173	0.46 (0.15–1.40)	0.72 (0.16–3.17)
> 1001 Birr	228	255	0.872	1.07 (0.46–2.53)	0.71 (0.22–2.31)
*History of cancer*					
No, I don’t have	226	280	1.0	1.0	1.0
Yes, I have	22	13	0.040	2.096 (1.03–4.26)	1.56 (0.62–3.94)

^**^Indicates *P*-value < 0.001 strongly associated, *indicates *P*-value < 0.05 weakly associated, 1.0 indicates reference

Women who have personal and family history of breast cancer were about 6 times [AOR: 6.06, (95% CI: 2.19–16.74)] more likely to perform BSE than women who do have not the history of breast cancer. A woman who has adequate knowledge of BSE about 3 times [AOR: 2.67 (95% CI: 1.18–6.04)] is more likely to perform BSE than women who have inadequate knowledge (Table 9).

**Table 9:** Bivariate and multivariate analysis of factors associated with the practice of BSE among women aged 20–70 at Gondar Town, Northwest Ethiopia, 2021

Variables	Practices of BSE		*P*-value	COR (95% CI)	AOR (95% CI)
	Good	Poor			
*Age of the women*					
20–29	30	63	1.0	1.0	1.0
30–39	28	49	0.574	1.20 (0.64–2.27)	0.92 (0.39–2.11)
40–49	11	24	0.929	0.96 (0.42–2.22)	0.93 (0.31–2.75)
50–70	8	35	0.103	0.48 (0.12–1.16)	0.47 (0.14–1.59)
*Marital status*					
Single	12	32	1.0	1.0	1.0
Married	51	106	0.511	1.28 (0.61–2.70)	1.56 (0.27–8.97)
Widowed	7	16	0.785	1.17 (0.39–3.54)	2.07 (0.41–10.42)
Divorced	7	17	0.868	1.20 (0.37–3.31)	1.56 (0.34–7.08)
*Level of education*					
Illiterate	5	17	1.0	1.0	1.0
Primary school	5	35	0.301	0.49 (0.12–1.91)	0.35 (0.07–1.78)
Secondary	16	66	0.739	0.82 (0.26–2.57)	0.88 (0.23–3.43)
College and above	51	53	0.030	3.27 (1.12–9.53)	3.15 (0.77–12.88)
*Occupation*					
Maid	5	15	1.0	1.0	1.0
Merchant	12	19	0.314	1.89 (0.55–6.57)	1.64 (0.33–8.09)
Student	6	20	0.880	0.90 (0.23–3.52)	0.76 (0.16–3.65)
Gov’t employee	29	29	0.058	3.00 (0.96–9.34)	2.75 (0.57–13.39)
Housewife	25	88	0.777	0.85 (0.28–2.57)	1.27 (0.28–5.78)
*Spouse educational level*					
Illiterate	5	12	1.0	1.0	1.0
Primary	5	15	0.764	0.80 (0.18–3.42)	0.68 (0.12–4.05)
Secondary	8	35	0.364	0.55 (0.15–2.00)	0.51(0.10–2.55)
College above	33	44	0.311	1.80 (0.58–5.61)	0.67 (0.15–2.92)
*History of cancer*					
No, I don’t have	59	154	1.0	1.0	1.0
Yes, I have	18	17	0.006	2.76 (1.34–5.72) *	6.06 (2.19–16.74) **
*Knowledge*					
Inadequate	13	67	1.0	1.0	1.0
Adequate	64	104	0.001	3.17 (1.62–6.20) **	2.67 (1.18–6.04) *
*Attitudes*					
Unfavorable	9	39	1.0	1.0	1.0
Favorable	68	132	0.044	2.23 (1.02–4.88)	1.99 (0.76–5.27)

^**^Indicates *P*-value < 0.001 strongly associated, *indicates *P*-value < 0.05 weakly associated, 1.0 indicates reference

## Discussion

The aim of this study was to assess knowledge, attitude, practice, and associated factors of breast self-examination among women in Gondar town. In this study, more than half 56% (n = 200) (95% CI: 52–62%) of the study participants had adequate knowledge regarding BSE, which is comparable with study conducted in Ethiopia that indicated 55.5% in Adwa town [24]. This similarities could be due to similar study subjects.

However, the finding is higher than that of Vietnam 22.7% [16], 34% Sudan [19], 41.5% Libya [18], 34.2% Arba Minch [32], 43.1% Jimma [23], and 25.6% Addis Ababa [21]. The difference might be due to by self-reporting data collection technique in Vietnam and Sudan, and the clustered sampling procedure was used in Libya. The self-reporting data collection method requires participants to respond to the researcher's questions without his/her interference [48]. In Ethiopia, the possible justification might be the educational level difference. In our study, 29.8% of participants were College and above educational status, but only 14.4% of study participants in Arba Minch, and 18.8% in Jima were college and above educational status. This finding is lower than the study conducted in Cameroon 78% [20], and 63.8% in Turkey [17]. The possible justification might be due to socio-economic, and study population differences. The current study was community-based, but that of Cameroon was conducted on patients attending a surgical outpatient clinic.

In this finding, about 46% (95% CI: 42–52%) of participants had a positive attitude towards breast self-examination, which is comparable with the study conducted in Ethiopia that indicated 46.3% in Adwa town [24].This consistency might be due to similar socio-cultural status and study population whose age were 20–70. However, it is lower than the studies conducted in Saudi Arabia 64.01% [25], 87.2% Pakistan [26], and 74.9% Libya [18]. This difference might be due to sampling size difference, educational level, and participant’s knowledge towards BSE, occupational difference, and information access availability.

In this finding, Nearly one-third 31% (n = 77) (95% CI: 25–37%) of participants had good practices towards BSE, which is comparable with the study done in Addis Ababa, 28.4% [33]. This consistency might be due to similar socio-cultural and economic status. Besides, factors that affect practice for early detection of BSE might be similar in both study settings. On contrary, this study is higher than the studies conducted in Arba Minch 13.3% [32], 12.1% Libya [18], and 8.5% Turkey [17]. The difference might be knowledge difference, and in Arba Minch, the majority (45%) of the participants are illiterate. On the other hand, this finding was lower than the studies conducted in Saudi Arabia 43% [27], and 37.6% in Ghana [29]. The possible justification might be educational and knowledge differences. In Saudi Arabia, the majority (65%) of participants had adequate knowledge about BSE, and 79% of the participant had an educational level completed university, whereas 88% of participants were aware of BSE in Ghana.

In this study, women’s educational level at College and University were about 4 times [AOR: 3.8, 95% CI: (1.43–10.14)] more likely to be knowledgeable with BSE than those who are illiterate. Also, the women whose husbands had an educational level College and University were about 3 times [AOR: 3.03, 95% CI: (1.04–8.84)] more likely to knowledgeable than those had illiterate husbands. Evidence shows that training women about breast self-examination goes correspondingly with their educational attainment of College level and above has a positive impact on their related knowledge and practices [49, 50].

Women’s College and above was also one of the significant factors for the attitudes of breast self-examination, women’s College and above was about 4 times AOR:4.18, (95% CI: 1.59–10.92) and secondary school was about 3 times [AOR: 2.80, 95% CI: (1.25–6.29)] more likely to had positive attitude towards BSE than women those who are illiterate. it was supported by the study done in Libya [18], and Turkey [17]. This might be due to educational attainment of secondary school and above could increase women’s attitudes towards breast self-examination besides it also enhance compliance towards breast self-examination behavior [51].

In this study, women who have a history of breast cancer were about 6 times [AOR: 6.06, 95% CI: (2.19–16.74)] more likely to perform BSE than their counter parts. This study was in line with the studies conducted in Jimma [23], Addis Ababa [36], and Libya [18]. A family history of breast cancer is positively affecting the practice of breast self-examination [52]. This could be women with a history of breast cancer performing breast self*-*examination at a regular basis and making them more cognizant*,* which in turn may lead to an earlier diagnosis of breast cancer*.*

## Limitations

As practice was not directly observed rather we used checklist that will not know how exactly they were practicing it. Other limitations were being conducted in a single urban community, which may not be representative of the rural community or other urban communities in Ethiopia.

## Conclusion

The findings of this study were considerable for inadequate knowledge, unfavorable attitude and poor practice towards BSE among women. Emphasis should be made to boost the attitude and practice of the women towards breast self-examination and strengthening of the implementation of comprehensive, systematic, and continuous BSE educational programs were recommended along with a breast cancer awareness campaign.

## Data Availability

The datasets used/or analyzed during the current study is available from the corresponding author on reasonable request.

## Abbreviations

ACS: American Cancer Society
BCA: Breast Cancer
BSE: Breast self-examination
CBE: Clinical breast examination
ECA: Ethiopian Cancer Association
SPSS: Statistical package social science
COR: Crude odds ratio
AOR: Adjusted odds ratio
CI: Confidence interval
UoG: University of Gondar
